# OncoOmics approaches to reveal essential genes in breast cancer: a panoramic view from pathogenesis to precision medicine

**DOI:** 10.1038/s41598-020-62279-2

**Published:** 2020-03-24

**Authors:** Andrés López-Cortés, César Paz-y-Miño, Santiago Guerrero, Alejandro Cabrera-Andrade, Stephen J. Barigye, Cristian R. Munteanu, Humberto González-Díaz, Alejandro Pazos, Yunierkis Pérez-Castillo, Eduardo Tejera

**Affiliations:** 1 Centro de Investigación Genética y Genómica, Facultad de Ciencias de la Salud Eugenio Espejo, Universidad UTE, Mariscal Sucre Avenue, Quito, 170129 Ecuador; 2 RNASA-IMEDIR, Computer Science Faculty, University of A Coruna, A Coruna, 15071 Spain; 3 Red Latinoamericana de Implementación y Validación de Guías Clínicas Farmacogenómicas (RELIVAF-CYTED), Quito, Ecuador; 4 Carrera de Enfermería, Facultad de Ciencias de la Salud, Universidad de Las Américas, Avenue de los Granados, Quito, 170125 Ecuador; 5 Grupo de Bio-Quimioinformática, Universidad de Las Américas, Avenue de los Granados, Quito, 170125 Ecuador; 6 Department of Chemistry, McGill University, 801 Sherbrooke Street West, Montreal, QC H3A 0B8 Canada; 7 Biomedical Research Institute of A Coruña (INIBIC), University Hospital Complex of A Coruna (CHUAC), A Coruna, 15006 Spain; 8 Centro de Investigación en Tecnologías de la Información y las Comunicaciones (CITIC), Campus de Elviña s/n, A Coruna, 15071 Spain; 9 Department of Organic Chemistry II, University of the Basque Country UPV/EHU, Leioa, 48940 Biscay Spain; 10 IKERBASQUE, Basque Foundation for Science, Bilbao, 48011 Biscay Spain; 11 Escuela de Ciencias Físicas y Matemáticas, Universidad de Las Américas, Avenue de los Granados, Quito, 170125 Ecuador; 12 Facultad de Ingeniería y Ciencias Agropecuarias, Universidad de Las Américas, Avenue de los Granados, Quito, 170125 Ecuador

**Keywords:** Breast cancer, Cancer genomics, Molecular medicine

## Abstract

Breast cancer (BC) is the leading cause of cancer-related death among women and the most commonly diagnosed cancer worldwide. Although in recent years large-scale efforts have focused on identifying new therapeutic targets, a better understanding of BC molecular processes is required. Here we focused on elucidating the molecular hallmarks of BC heterogeneity and the oncogenic mutations involved in precision medicine that remains poorly defined. To fill this gap, we established an OncoOmics strategy that consists of analyzing genomic alterations, signaling pathways, protein-protein interactome network, protein expression, dependency maps in cell lines and patient-derived xenografts in 230 previously prioritized genes to reveal essential genes in breast cancer. As results, the OncoOmics BC essential genes were rationally filtered to 140. mRNA up-regulation was the most prevalent genomic alteration. The most altered signaling pathways were associated with basal-like and Her2-enriched molecular subtypes. *RAC1*, *AKT1*, *CCND1*, *PIK3CA*, *ERBB2*, *CDH1*, *MAPK14*, *TP53*, *MAPK1*, *SRC*, *RAC3*, *BCL2*, *CTNNB1*, *EGFR*, *CDK2*, *GRB2*, *MED1* and *GATA3* were essential genes in at least three OncoOmics approaches. Drugs with the highest amount of clinical trials in phases 3 and 4 were paclitaxel, docetaxel, trastuzumab, tamoxifen and doxorubicin. Lastly, we collected ~3,500 somatic and germline oncogenic variants associated with 50 essential genes, which in turn had therapeutic connectivity with 73 drugs. In conclusion, the OncoOmics strategy reveals essential genes capable of accelerating the development of targeted therapies for precision oncology.

## Introduction

Breast cancer (BC) is a complex and heterogeneous disease characterized by an intricate interplay between different biological aspects such as ethnicity, genomic alterations, gene expression deregulation, hormone disruption, signaling pathway alterations, hypoxia, and environmental determinants^1,2^. Over the last years, prevention, treatment and survival strategies have evolved favorably; however, there are BC profiles that remain incurable^3^. Nowadays, BC is the leading cause of cancer-related death among women (627,000; 15% cases) and the most commonly diagnosed cancer (2,088,849; 24% cases) worldwide^4^.

The development of large-scale DNA sequencing, gene expression, proteomics, large-scale RNA interference (RNAi) screens, large-scale CRISPR-Cas9 screens and patient-derived xenografts (PDXs) has allowed us to better understand the molecular landscape of oncogenesis. Considerable progress has been made in discovering coding and non-coding somatic drivers^5,6^, cancer driver genes^7,8^, cancer driver mutations^9,10^, germline variants^11^, driver fusion genes^12,13^, alternatively spliced transcripts^14^, expression-based stratification^15^, molecular subtyping^16^, biomarkers^17^, druggable enzymes^18^, cancer dependencies^19–22^, and drug resistance^23^.

Scientific advances made to date mark the era called the “end of the beginning” of cancer omics. In other words, each approach that was previously mentioned needs to be fully understood as a part of a complex network, analyzing the mechanistic interplay of signaling pathways, protein-protein interactome (PPi) networks, enrichment maps, gene ontology (GO), deep learning, molecular dependencies and genomic alterations per intrinsic molecular subtype: basal-like (estrogen receptor (ER)^−^, progesterone receptor (PR)^−^, human epidermal growth factor receptor 2 (Her2)^−^, cytokeratin 5/6^+^ and/or EGFR^+^); Her2-enriched (ER^−^, PR^−^, Her2^+^); luminal A (ER^+^ and/or PR^+^, Her2^−^, low Ki67); luminal B with Her2^−^ (ER^+^ and/or PR^+^, Her2^−^, low Ki67); luminal B with Her2^+^ (ER^+^ and/or PR^+^, Her2^+^, any Ki67); and normal like^24–30^.

Here we focus on elucidating the molecular hallmarks of BC essential genes and the oncogenic mutations applied in precision medicine that remains poorly defined. To fill this gap, we propose the OncoOmics strategy that consists in the analysis of genomic alterations (mRNA up-regulation, mRNA down-regulation, putative driver mutation, copy number variant (CNV) amplification, CNV deep deletion, and fusion gene), signaling pathways, PPi network, protein expression, BC dependencies in cell lines and patient-derived xenografts in a set of previously prioritized genes. These genes will come from our Consensus Strategy (CS) study^29^, the Pan-Cancer Atlas (PCA) project^3,13,31–37^, the Cancer Genome Interpreter (CGI) study^38^, and the Pharmacogenomics Knowledgebase (PharmGKB)^39^.

In our previous studies, López-Cortés *et al*., Tejera *et al*., and Cabrera-Andrade *et al*., developed a Consensus Strategy that was proved to be highly efficient in the recognition of gene-disease association^29,40,41^. The main objective was to apply several bioinformatics methods to explore BC pathogenic genes. On the other hand, The Cancer Genome Atlas (TCGA) has concluded the most sweeping cross-cancer analysis yet undertaken, namely the PCA project^32^. PCA reveals how genomic alterations and protein expression collaborate in BC progression, providing insights to prioritize the development of new treatments^3,13,31–37^. The CGI flags genomic biomarkers of drug response with different levels of clinical relevance^38^. Lastly, PharmGKB is a comprehensive resource that curates and spreads knowledge of the impact of clinical annotations on drug response^39,42^. PharmGKB collects the precise guidelines for the application of precision medicine and pharmacogenomics in clinical practice published by the European Society for Medical Oncology (ESMO), the National Comprehensive Cancer Network (NCCN), the Royal Dutch Association for the Advancement of Pharmacy (DPWG), the Canadian Pharmacogenomics Network for Drug Safety (CPNDS) and the Clinical Pharmacogenetics Implementation Consortium (CPIC)^43–46^. Hence, we identified essential genes, oncogenic mutations and potential therapeutic targets that could be incorporated into strategies aimed at improving novel drug development and precision medicine in BC.

## Results

### OncoPrint of genomic alterations according to the Pan-Cancer Atlas

PCA has reported the clinical data of 1084 individuals with BC and it can be visualized in the Genomic Data Commons of the National Cancer Institute and in the cBioPortal^47,48^. In regard to molecular subtypes and tumor stages, 46% were lumina A, 18% luminal B, 7% Her2-enriched, 16% basal-like and 3% normal-like, whereas 17% were tumor stage 1 (T1), 58% T2 stage, 23% T3 stage and 2% T4 stage (Supplementary Table S1).

**Figure 1:**
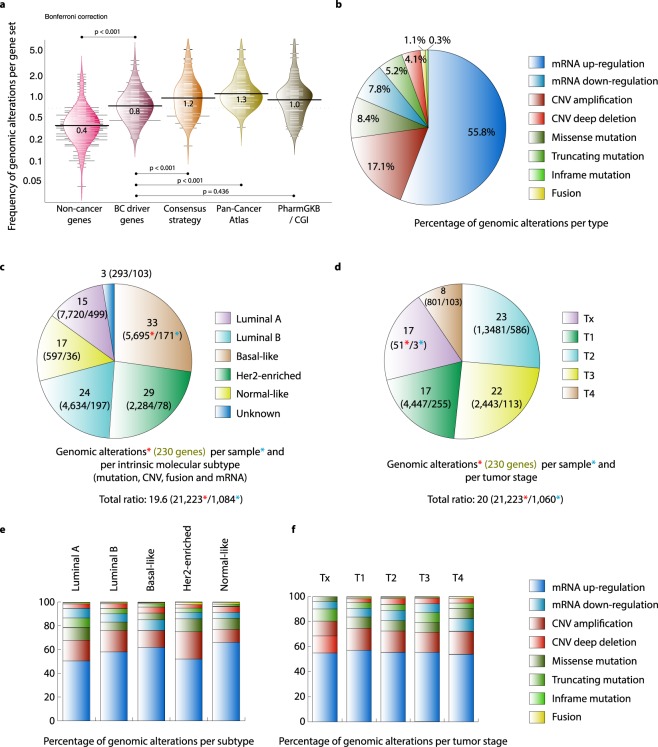
Genomic alterations of the breast cancer cohort according to PCA. (**a**) Frequency of genomic alterations per gene set (non-cancer genes, BC driver genes according to the Network of Cancer Genes, Consensus Strategy, BC genes according to PCA, BC biomarkers according to the PharmGKB and CGI). Bonferroni correction with significant level of P < 0.05 and a 95% confidence interval was performed. (**b**) Percentage of genomic alterations per type. (**c**) Ratio of genomic alterations per intrinsic molecular subtype. (**d**) Ratio of genomic alterations per tumor stage. (**e**) Percentage of genomic alterations per type and molecular subtype. (**f**) Percentage of genomic alterations per type and tumor stage.

Figure 1a shows the frequency mean of genomic alterations per gene set. The frequency mean of the PCA gene set was 1.3, followed by the CS gene set (1.2), the PharmGKB/CGI gene set (1.0), BC driver genes (0.8), and non-cancer genes (0.4) (Supplementary Table S2). Consequently, we performed a multiple comparison of the genomic alteration frequencies using the Bonferroni correction in order to determine statistical significance among gene sets. There were significant differences between BC driver genes and non-cancer genes (P < 0.001), the PCA gene set and BC driver genes (P < 0.001), and the CS gene set and BC driver genes (P < 0.001). Hence, the fact that gene sets of interest (CS and PCA) presented significant differences in the amount of genomic alterations versus BC driver genes could indicate that we are analyzing potentially essential genes in BC. Figure 1b shows the percentage of genomic alterations per type. The most common genomic alterations were mRNA up-regulation (55.8%), CNV amplification (17.1%), and missense mutations (8.4%). Figure 1c shows the ratio of genomic alterations in the 230 genes per sample and molecular subtype. Basal-like had the highest ratio (n = 33), followed by Her2-enriched (29), luminal B (24), normal-like (17), and luminal A (15). The ratio of all BC samples was 19.6. Figure 1d shows the ratio of genomic alterations in the 230 genes per sample and tumor stage. T2 stage had the highest ratio (23), followed by T3 (22), T1 (17) and T4 (8). Figure 1e,f show the percentage of genomic alterations per subtype and tumor stage, respectively. mRNA up-regulation and CNV amplification were the most common alterations in all molecular subtypes and tumor stages.

**Figure 2:**
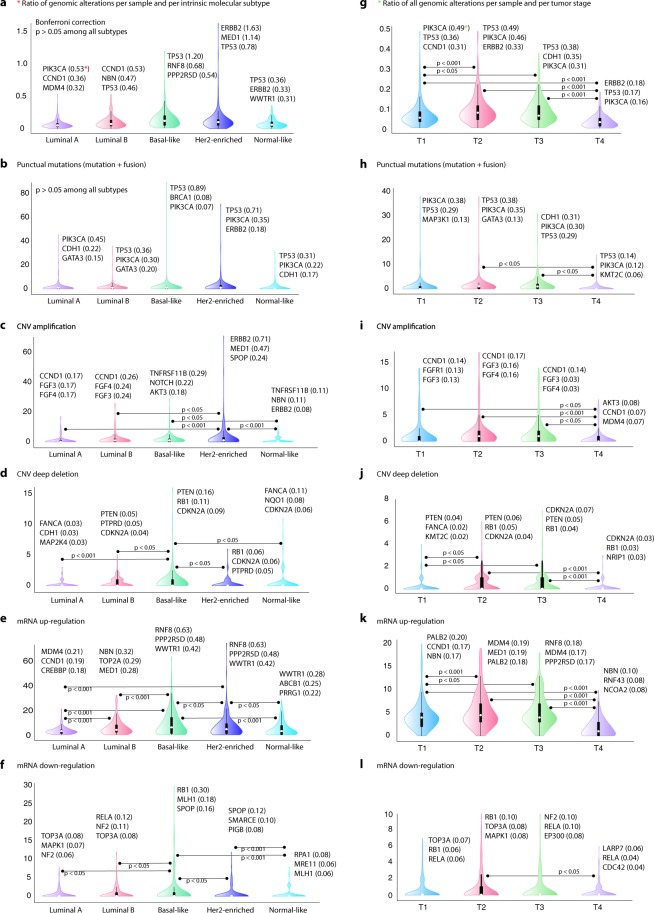
Ranking of genes with the highest amount of genomic alterations per molecular subtype and tumor stage. (**a**) Frequency of genomic alterations (punctual mutations, copy number variants and mRNA expression) per molecular subtype. (**b**) Frequency of genomic alterations per tumor stage. (**c**) Frequency of punctual mutations per molecular subtype. (**d**) Frequency of punctual mutations per tumor stage. (**e**) Frequency of CNV amplifications per molecular subtype. (**f**) Frequency of CNV amplifications per tumor stage. (**g**) Frequency of CNV deep deletions per molecular subtype. (**h**) Frequency of CNV deep deletions per tumor stage. (**i**) Frequency of mRNA up-regulation per molecular subtype. (**j**) Frequency of mRNA up-regulation per tumor stage. (**k**) Frequency of mRNA down-regulation per molecular subtype. (**L**) Frequency of mRNA down-regulation per tumor stage.

Figure 2 shows the ranking of genes with the highest amount of genomic alterations per molecular subtype and tumor stage. Regarding molecular subtypes, *PIK3CA* was the most altered gene in luminal A, *CCND1* in luminal B, *TP53* in basal-like and normal-like, and *ERBB2* in Her2-enriched (Fig. 2a). Figure 2b–f show genes with the highest ratio of mutations, CNV amplifications, CNV deep deletions, mRNA up-regula tions, and mRNA down-regulations per molecular subtype (Tables S3–S7). After Bonferroni  correction, we obtained statistically significant differences (P < 0.05) regarding CNV amplifications, CNV deep deletions, mRNA up-regulations, and mRNA down-regulations among molecular subtypes. On the other hand, the most altered genes per tumor stage were *PIK3CA* in T1 stage, *TP53* in T2 and T3, and *ERBB2* in T4 (Fig. 2g). Figure 2h–l show genes with the highest percentage of mutations, CNV amplifications, CNV deep deletions, mRNA up-regulations, and mRNA down-regulations per tumor stage (Tables S8–S12). We found statistically significant differences (P < 0.05) regarding all genomic alterations among tumor stages using the Bonferroni correction test.

**Figure 3:**
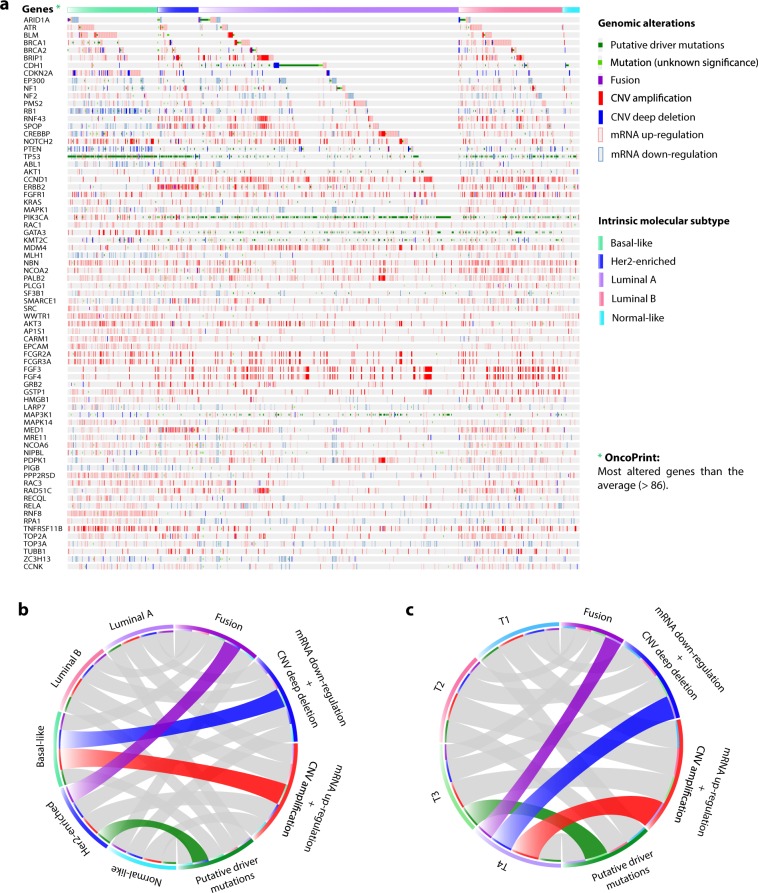
OncoPrint of genomic alterations according to the Pan-Cancer Atlas. (**a**) OncoPrint of genes with more genomic alterations than the average (>86) per molecular subtype. (**b**) Circos plot between molecular subtypes and the highest amount of genomic alterations (fusion genes, mRNA down-regulation plus CNV deep deletion, mRNA upregulation plus CNV amplification, and driver mutations). (**c**) Circos plot between tumor stages and the highest amount of genomic alterations.

The first OncoOmics approach was focused on genes with the highest amount of genomic alterations (more than the average). The panoramic landscape of genomic alterations was termed OncoPrint and is shown in Fig. 3a. Putative driver mutations were taken into account for this analysis, discarding passenger mutations (Figure S1 and Supplementary Table S13). Figure 3b,c show circos plots of interactions among molecular subtypes, tumor stages, and genomic alterations of the most altered genes (Supplementary Table S14). Highest amount of fusion genes were in Her2-enriched subtype and T4 stage, highest amount of mRNA down-regulation + CNV deep deletion were in basal-like subtype and T4 stage, highest amount of mRNA up-regulation + CNV amplification were in basal-like subtype and T4 stage, lastly, highest amount of putative driver mutations were in Her2-enriched subtype and T3 stage. As result, the first OncoOmics approach reveled 73 essential genes with highest frequencies of genomic alterations.

### Pathway enrichment analysis

**Figure 4:**
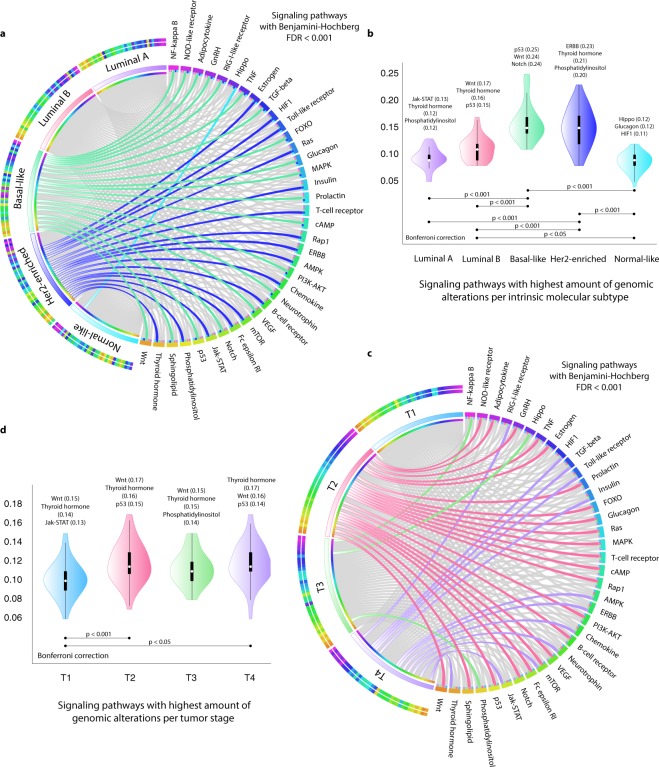
Pathway enrichment analysis per molecular subtype and tumor stage. (**a**) Circos plot between molecular subtypes and the most altered signaling pathways. (**b**) Violin plots showing the frequency of the most altered signaling pathways per molecular subtype. (**c**) Circos plot between tumor stages and the most altered signaling pathways. (**d**) Violin plots showing the frequency of the most altered signaling pathways per tumor stage.

This enrichment analysis was performed using David Bioinformatics Resource to obtain integrated information from the Kyoto Encyclopedia of Genes and Genomes (KEGG)^49–52^. The enrichment analysis of signaling pathways was carried on in the 230 genes, obtaining more than 50 terms with a Benjamini-Hochberg - false discovery rate (FDR) <0.01 (Supplementary Table S15). Subsequently, genomic alterations of genes that make up each signaling pathway were analyzed according to the molecular subtype and tumor stage. Figure 4a shows a circos plot correlating molecular subtypes with signaling pathways (Supplementary Table S16). NF-kappa ß, NOD-like receptor, adipocytokine, GnRH, RIG-like receptor, TNF, TGFß, FOXO, glucagon, MAPK, prolactin, cAMP, PI3K-AKT, neurotrophin, VEGF, notch, p53, sphingolipid and Wnt signaling pathways were more altered in basal-like; estrogen, HIF1, toll-like receptor, ras, insulin, T-cell receptor, rap1, ERBB, AMPK, chemokine, B-cell receptor, mTOR, Fc-epsilon RI, Jak-STAT, phosphatidylinositol and thyroid hormone pathways were more altered in Her2-enriched; and Hippo pathway in normal-like. On the other hand, Fig. 4b shows the ranking of the most altered signaling pathways per molecular subtype. Jak-STAT pathway was more altered in luminal A; Wnt pathway in luminal B; p53 pathway in basal-like; ERBB pathway in Her2-enriched; and Hippo pathway in normal-like (Supplementary Table S17). After Bonferroni correction, we observed statistically significant differences (P < 0.001) regarding the amount of genomic alterations in signaling pathways among molecular subtypes.

Figure 4c shows a circos plot correlating tumor stages with signaling pathways according to the frequency of genomic alterations (Supplementary Table S16). NOD-like receptor, adipocytokine, GnRH, TNF, estrogen, prolactin, FOXO, glucagon, ras, MAPK, T-cell receptor, cAMP, rap1, PI3K-AKT, B-cell receptor, VEGF, mTOR, Fc epsilon RI, NOTCH, p53, sphingolipid and Wnt pathways were more altered in stage T2; NF-kappa ß, Hippo and phosphatidylinositol pathways were more altered in T3 stage; and RIG-like receptor, HIF1, TGFß, toll-like receptor, insulin, AMPK, ERBB, chemokine, neurotrophin, mTOR, jak-STAT and thyroid hormone pathways were more altered in T4 stage. On the other hand, Fig. 4d shows the ranking of the most altered signaling pathways per tumor stage. Wnt pathway was more altered in T1, T2 and T3 stages; and thyroid hormone pathway was more altered in T4 stage (Supplementary Table S18). We found statistically significant differences (P < 0.001) regarding the amount of genomic alterations in signaling pathways among different tumor stages using the Bonferroni correction test.

### Protein-protein interactome network

**Figure 5:**
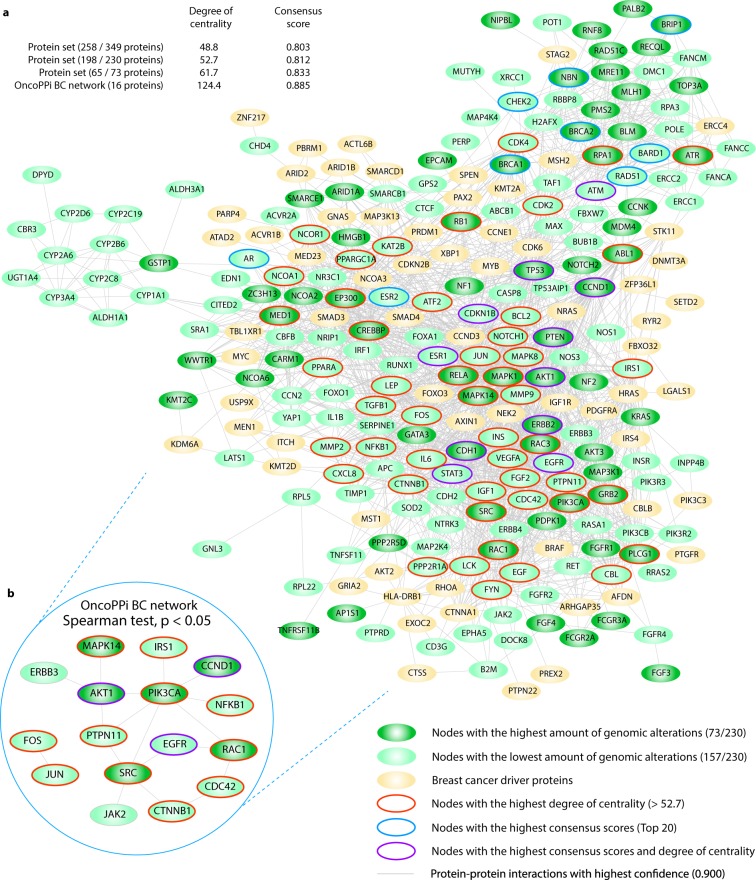
Protein-protein interactome network. (**a**) Network composed of BC driver genes and genes of our study (PCA gene set, consensus strategy gene set and PharmGKB gene set. (**b**) Significant correlation (P < 0.05) of degree centrality and consensus score between the OncoPPi BC network and our String PPi network.

The second OncoOmics approach was focused on proteins with the highest degree centrality and consensus score in the String PPi network. The PPi network was performed to better understand BC behavior using the String Database and Cytoscape^53,54^. With the indicated cutoff of 0.9, the final interactome network had 258 nodes conformed by 198 (86%) proteins from the CS, PCA and PharmGKB/CGI sets. Regarding nodes with the highest amount of genomic alterations showed previously in the OncoPrint, 65 (89%) of them integrated this network (Fig. 5a). On the other hand, out of the 258 proteins that make up our String PPi network, 16 (6%) proteins and 18 edges were part of the OncoPPi BC network^55,56^. The degree centrality made it possible to establish a significant correlation (Spearman test, P < 0.05) between our String PPi network and the OncoPPi BC network (Fig. 5b).

Considering degree centrality and consensus scores from our previous study^29^, there was enrichment among sub-networks (Fig. 5a,b). The degree centrality average in the whole network was 48.8, and out of the OncoPPi BC network was 124.4. Meanwhile, the average of consensus score of the whole network was 0.803, and out of the OncoPPi BC network was 0.885. As result, the second OncoOmics approach reveled 40 proteins with both the highest degree centrality and consensus score, as shown in Supplementary Table S19.

### Protein expression analysis

**Figure 6:**
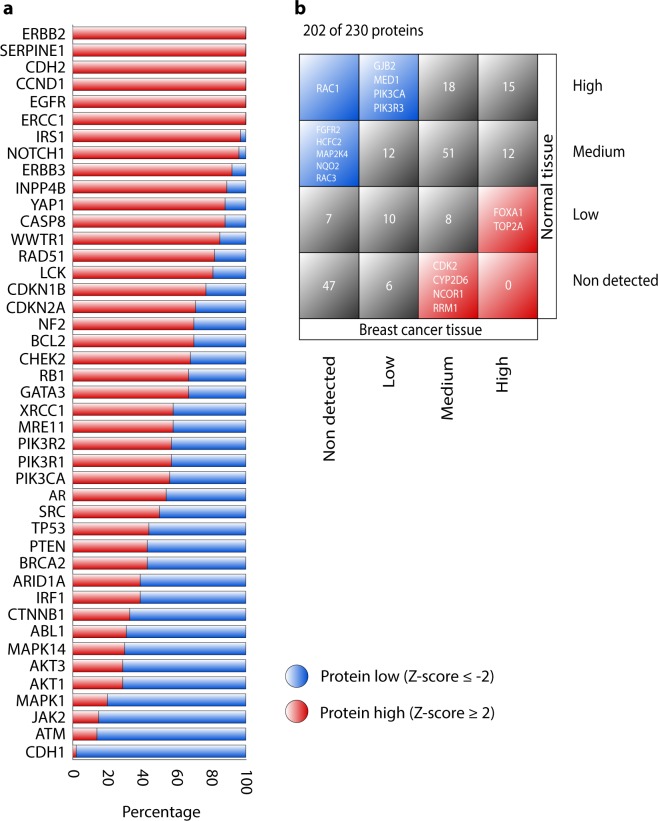
Protein expression analyses. (**a**) Proteins (n = 43) with alterations in the expression levels. Low expression proteins with Z-score ≤ −2 and high expression proteins with Z-score ≥ 2 according to TCGA. (**b**) Comparison of protein expression levels (n = 202) by immunohistochemistry between BC tissue and normal tissue according to The Human Protein Atlas.

The third OncoOmics approach was focused on proteins with considerable high and low expressions in BC. Figure 6a shows 43 proteins with significant high expression (Z-scores **≥** 2) and low expression (Z-scores ≤ −2) analyzed with the reverse-phase protein array (RPPA) and mass spectrometry, in a cohort of 994 individuals according to TCGA (Supplementary Table S20). On the other hand, the Human Protein Atlas (HPA) presented a map of the human tissue proteome based on tissue microarray-based immunohistochemistry. HPA has analyzed 202 (88%) of the 230 proteins of our study, classifying the protein expression in high, medium, low and non-detected. As results, RAC1, GJB2, MED1, PIK3CA, PIK3R3, FGFR2, HCFC2, MAP2K4, NQO2 and RAC3 were proteins with high/medium expression in normal tissue, and low/non-detected expression in BC tissue. Meanwhile, CDK2, CYP2D6, NCOR1, RRM1, FOXA1 and TOP2A were proteins with hi gh/medium expression in BC tissue, and low/non-detected expression in normal tissue (F ig. 6b and Supplementary Table S21)^57,58^. As result, the third OncoOmics approach revealed 60 proteins with significant altered expression levels as shown in Tables S20 and S21.

### Breast cancer dependency map

**Figure 7:**
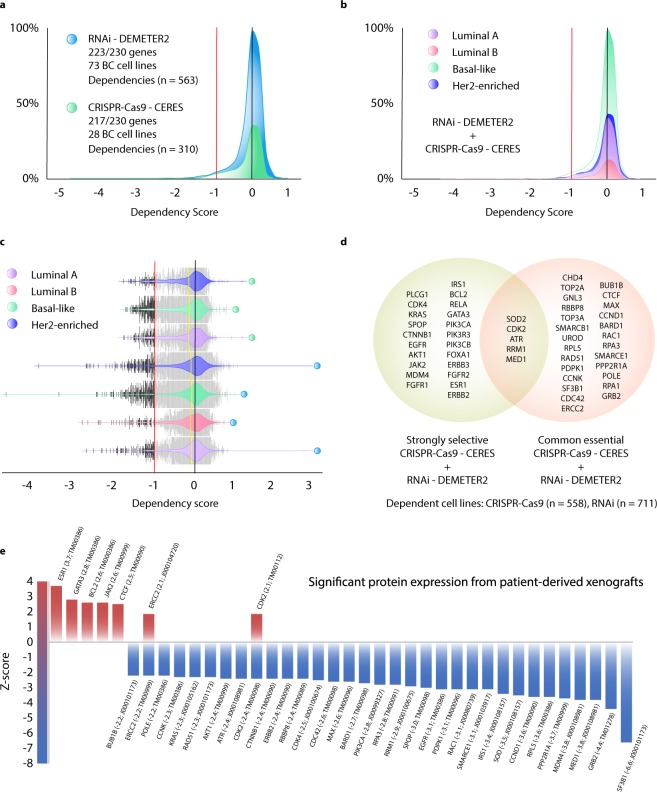
BC dependency maps in cell lines and patient-derived xenografts. (**a**) Dependency score of gene sets using RNAi DIMETER2 and CRISPR-Cas9 CERES algorithms in BC cell lines. (**b**) Dependency score of BC gene sets per molecular subtypes. (**c**) Violin plots of dependencies per molecular subtypes. All substantial dependencies < −1 are in black. (**d**) Venn diagram of strongly selective and common essential genes in all cancer cell lines. (**e**) Significant protein expression from patient-derived xenografts.

The first analysis of the fourth OncoOmics approach consisted in identifying genes that are essential for breast cancer cell proliferation and survival performing systematic loss-of-function screens in a large number of well-annotated cell lines representing the tumor heterogeneity^19–22^. Figure 7a shows the distribution of dependency scores of 227 genes through DEMETER2, an analytical framework for analyzing genome-scale RNAi loss-of-function screens in 73 BC cell lines (Supplementary Table S22). Our results showed 563 dependencies with at least one score ≤ −1 in 57 (25%) essential genes. At the same time, Fig. 7a shows the distribution of dependency scores of 217 genes through CERES, an analytical framework for analyzing genome-scale CRISPR-Cas9 loss-of-function screens in 28 BC cell lines (Supplementary Table S23). Our results showed 310 dependencies with at least one score ≤ −1 in 34 (16%) essential genes. Figure 7b shows the distribution of dependency scores of DEMETER2 and CERES per molecular subtype. The genome-scale RNAi loss-of-function screens detected 165 (29%) dependencies in 19 Her2-enriched cell lines (ratio = 8.7), 110 (20%) in 13 luminal A cell lines (8.5), 57 (10%) in 7 luminal B cell lines (8.1), and 231 (41%) in 34 basal-like cell lines (6.8), whereas the genome-scale CRISPR-Cas9 loss-of-function screens detected 85 (27%) dependencies in 7 luminal A cell lines (ratio = 12.1), 176 (15%) in 16 basal-like cell lines (11), and 49 (16%) in 5 Her2-enriched cell lines (9.8). Figure 7c shows violin plots of dependencies per molecular subtype. DEMETER2 has detected a greatest number of substantial dependencies in basal-like, followed by Her2-enriched, luminal A and luminal B, whereas CERES has detected a greatest number of substantial dependencies in basal-like, followed by luminal A and Her2-enriched. Figure 7d shows a Venn diagram of 22 strongly selective genes, 26 common essential genes, and 5 strongly selective and common essential genes in breast and other cancer cell lines.

### Patient-derived xenografts

The second analysis of the fourth OncoOmics approach consisted in identifying proteins with significant expression in PDXs. According to Woo *et al*., PDXs are *in vivo* models of human cancer that are useful for translational cancer research and therapy selection for individual patient. We analyzed the 66 strongly selective and common essential genes of BC cell lines using the Jackson Laboratory PDX resource^59^. Figure 7e shows 7 proteins with significant high expression (Z-score **≥** 2) and 33 proteins with significant low expression (Z-scores ≤ −2) with its respective mice model ID. As result, the fourth OncoOmics approach revealed 38 proteins with significant expression in both BC cell lines and patient-derived xenografts (Supplementary Tables S22 and S23).

### OncoOmics approaches to reveal essential genes in BC

**Figure 8:**
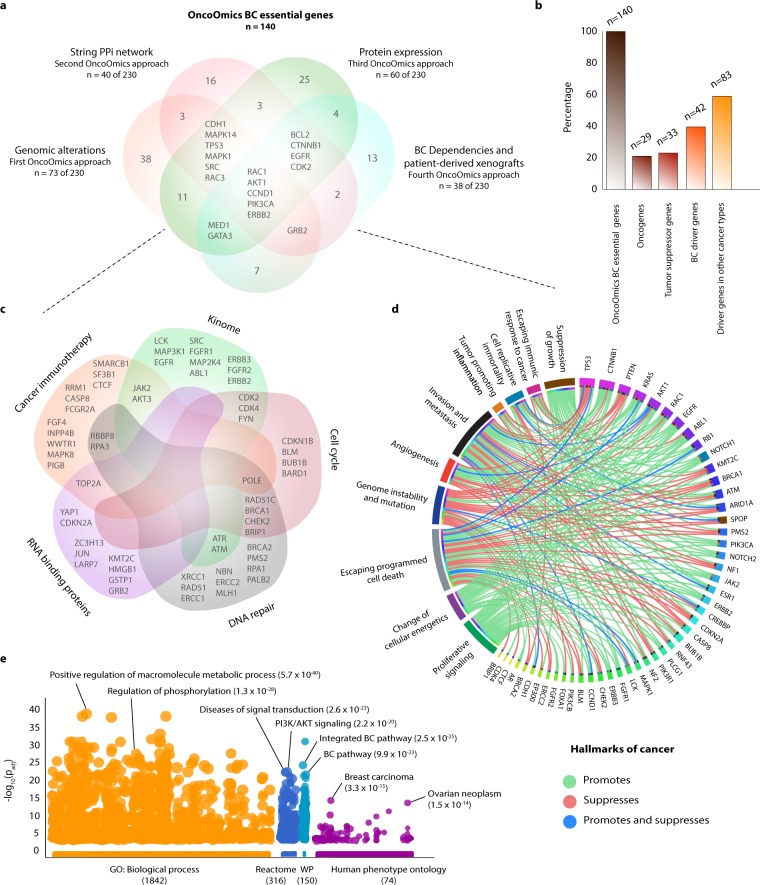
OncoOmics BC essential genes. (**a**) Venn diagram of the most essential genes per genomics approach (genomic alterations, String PPi network, protein expression, and BC dependencies/patient-derived xenografts). (**b**) Percentage of oncogenes, tumor suppressor genes and driver genes in other cancer types. (**c**) Venn diagram of the most essential genes related to cancer immunotherapy, kinome signaling, cell cycle, DNA repair and RNA-binding proteins. (**d**) Circos plot of genes with hallmarks of cancer. (**e**) Most significant g:Profiler features of the OncoOmics BC essential genes according to GO: biological processes, Reactome pathways, WikiPathways and the human phenotype ontology.

After analyses of the four OncoOmics approaches (genomic alterations, String PPi network, protein expression and BC dependencies/patient-derived xenografts), we used a Venn diagram to integrate essential genes, termed OncoOmics BC essential genes. Consequently, we could observe 140 essential genes in at least one OncoOmics approach; of them, 92 were essential in one OncoOmics approach, 30 were essential in two OncoOmics approaches, 13 were essential in three OncoOmics approaches, and 5 were essential in all OncoOmics approaches as shown in Fig. 8a and Supplementary Table S24.

The 140 OncoOmics BC essential genes were conformed by oncogenes (21%), tumor suppressor genes (24%) and driver genes in other cancer types (59%)^60^ (Fig. 8b). Additionally, some of these OncoOmics BC essential genes were involved in cancer immunotherapy^61^, kinome signaling^62^, cell cycle^63^, DNA repair^64^ and RNA-binding as shown in Fig. 8c and Supplementary Table S25^65^.

Figure 8d shows a circos plot detailing the correlation between 48 (34%) OncoOmics BC essential genes and hallmarks of cancer. Suppression of growth was promoted by *AKT1*, *CTNNB1*, *PTEN*, *RB1* and *TP53*; escaping immune response to cancer was promoted by *CTNNB1*, *EGFR* and *RAC1*; cell replicative immortality was promoted by *CTNNB1*, *KRAS* and *NOTCH1*; tumor promoting inflammation was promoted by *KRAS*; metastasis was promoted by *ABL1*, *CTNNB1*, *EGFR*, *KRAS*, *RAC1* and *RB1*; angiogenesis was promoted by *ABL1*, *CTNNB1*, *EGFR*, *KRAS*, *NOTCH1* and *RAC1*; genome instability was promoted by *ABL1* and *RB1*; escaping programmed cell death was promoted by *AKT1*, *CTNNB1*, *EGFR*, *NOTCH1*; change of cellular energetics was promoted by *ABL1*, *AKT1*, *CTNNB1*, *EGFR*, *KRAS*, *NOTCH1*, *PTEN*, *RB1* and *TP53*; finally, proliferative signaling was promoted by *ABL1*, *AKT1*, *CTNNB1*, *EGFR*, *KRAS*, *NOTCH* and *RAC1* (Supplementary Table S26).

### Enrichment map of the OncoOmics BC essential genes

**Figure 9:**
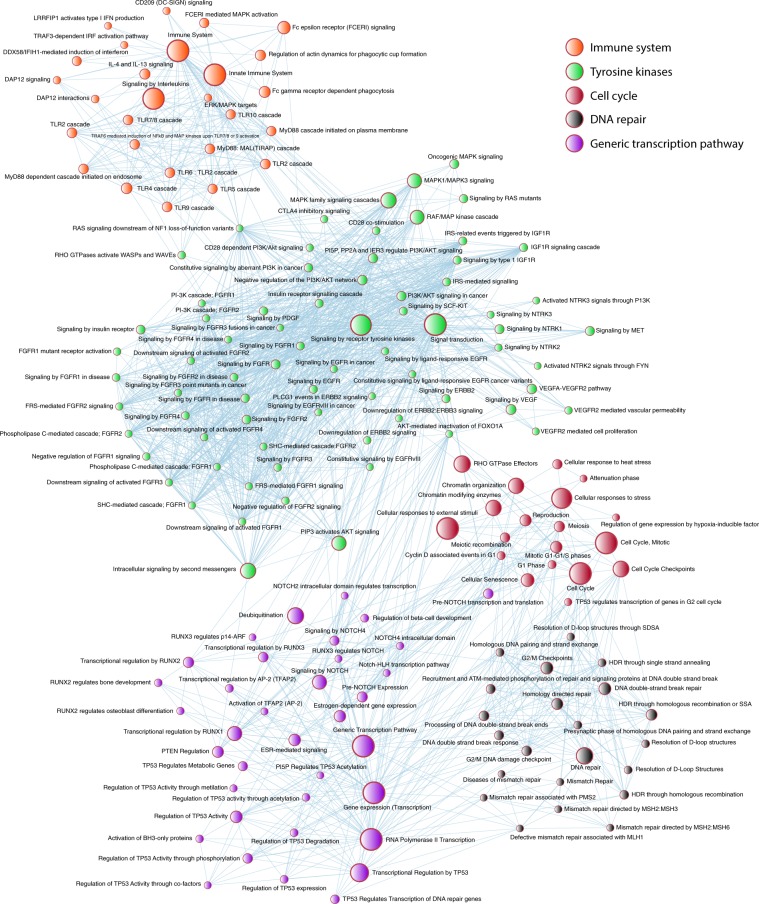
Pathway enrichment analysis of the OncoOmics BC essential genes using g:Profiler and EnrichmentMap. Most significant Reactome pathways related to immune system, kinome signaling, cell cycle, DNA repair and genetic transcription.

Figure 8e shows the enrichment map of the 140 OncoOmics BC essential genes. g:Profiler searches for a collection of genes representing GO terms, pathways and disease phenotypes^66^. The most significant GO: biological processes with a FDR < 0.001 was positive regulation of macromolecule metabolic process (Supplementary Table S27); the most significant GO: molecular function was phosphatidylinositol 3-kinase activity (Supplementary Table S28); the most significant Reactome pathway was generic transcriptor pathway (Supplementary Table S29)^67^; additionally, the most relevant disease, according the Human Phenotype Ontology, was breast carcinoma (Supplementary Table S30)^68^. Subsequently, g:Profiler annotations were analyzed with the EnrichmentMap software and visualized using Cytoscape, in order to generate network interactions of the most relevant GO: biological processes (Supplementary Fig. S2) and Reactome pathways (Fig. 9) related to immune system, tyrosine kinase, cell cycle and DNA repair pathways^54,66^.

### Clinical trials

**Figure 10:**
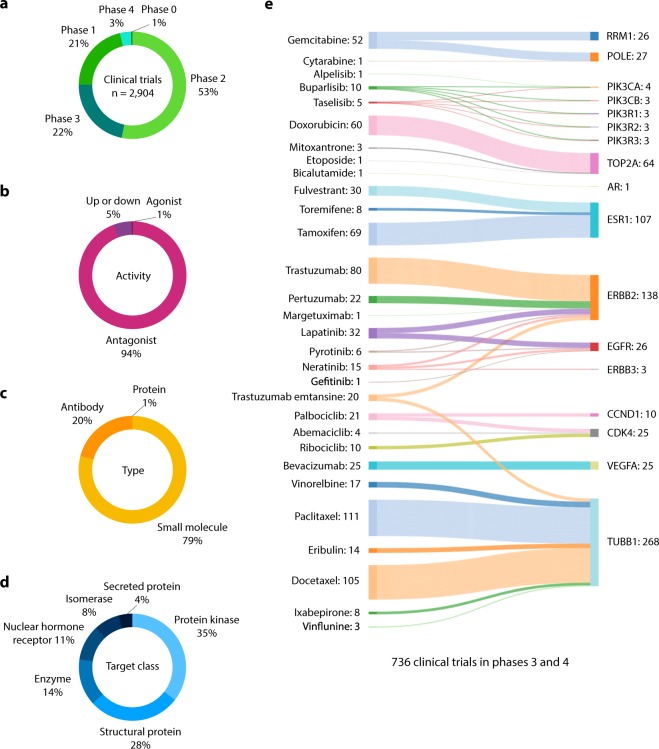
Current status of clinical trials in the OncoOmics BC essential proteins. (**a**) Clinical trials   per phase. (**b**) Clinical trials per activity. (**c**) Clinical trials per type. (**d**) Clinical trials per target class. (**e**) Correlation of drugs with proteins in advanced stages of clinical trials (3 and 4) using a Sankey plot.

Figure 10 and Supplementary Table S31 details the current status of clinical trials regarding OncoOmics BC essential proteins, according to the Open Targets Platform^69^. There are 98 drugs that are being analyzed in 2,904 clinical trials in 28 of 140 OncoOmics BC essential proteins (Fig. 10a). The top 10 drugs with the highest number of clinical trials in process or completed were paclitaxel (370), trastuzumab (315), docetaxel (262), doxorubicin (204), gemcitabine (196), lapatinib (152), tamoxifen (131), fulvestrant (129), bevacizumab (120) and neratinib (110). Regarding drugs, 94% were antagonists, 79% were small molecules, and 35% were protein kinases as shown in Fig. 10b–d, respectively. Additionally, drugs with the highest number of clinical trials in phases 3 and 4 were paclitaxel (111), docetaxel (105), trastuzumab (80), tamoxifen (69) and doxorubicin (60) as shown in a Sankey plot detailed in Fig. 10e.

### Precision medicine

Precision oncology focuses on matching the most effective and safe treatment based on the ‘omics’ profile of each individual or population^70,71^. However, the identification of driver mutational events remains the biggest challenge^72^. There are some consortiums and studies that have robustly identified variants associated with BC. Tamborero *et al*. detailed a compendium of 62 somatic and 398 germline validated oncogenic mutations in 14 OncoOmics BC essential genes (Supplementary Table S32)^38^. Huang *et al*. identified 87 pathogenic germline variants in 22 OncoOmics BC essential genes^73^ (Supplementary Table S33). Long *et al*.^74,75^, Cai *et al*.^76^, Michailidou *et al*.^77^, and the Breast Cancer Association Consortium performed genome-wide association studies identifying 172 germline variations related to BC development (Supplementary Table S34). The Precision Medicine Knowledgebase (PreMedKB) detailed a compendium of 2791 germline variants in 7 OncoOmics BC essential genes (Supplementary Table S35)^71^. PharmGKB enriched clinical guidelines with 59 well-known clinical annotations related to 29 OncoOmics BC essential genes (Supplementary Table S36)^42,78,79^. Finally, the Pan-Cancer Analysis of Whole Genomes (PCAWG) Consortium identified 19 non-coding somatic mutations and 17 coding somatic mutations in BC (Supplementary Table S37)^6^.

Regarding the Ensembl Variant Effect Predictor^80^, 1,102 of 3,565 variants were processed, being 24% intron variants, 16% missense variants, 15% downstream gene variants, 10% stop gained, 7% upstream gene variants, 7% NMD transcript variants, 4% splice region variants, 4% 3′ untranstaled region variants, and 2% splice acceptor variants (Supplementary Table S38).

**Figure 11:**
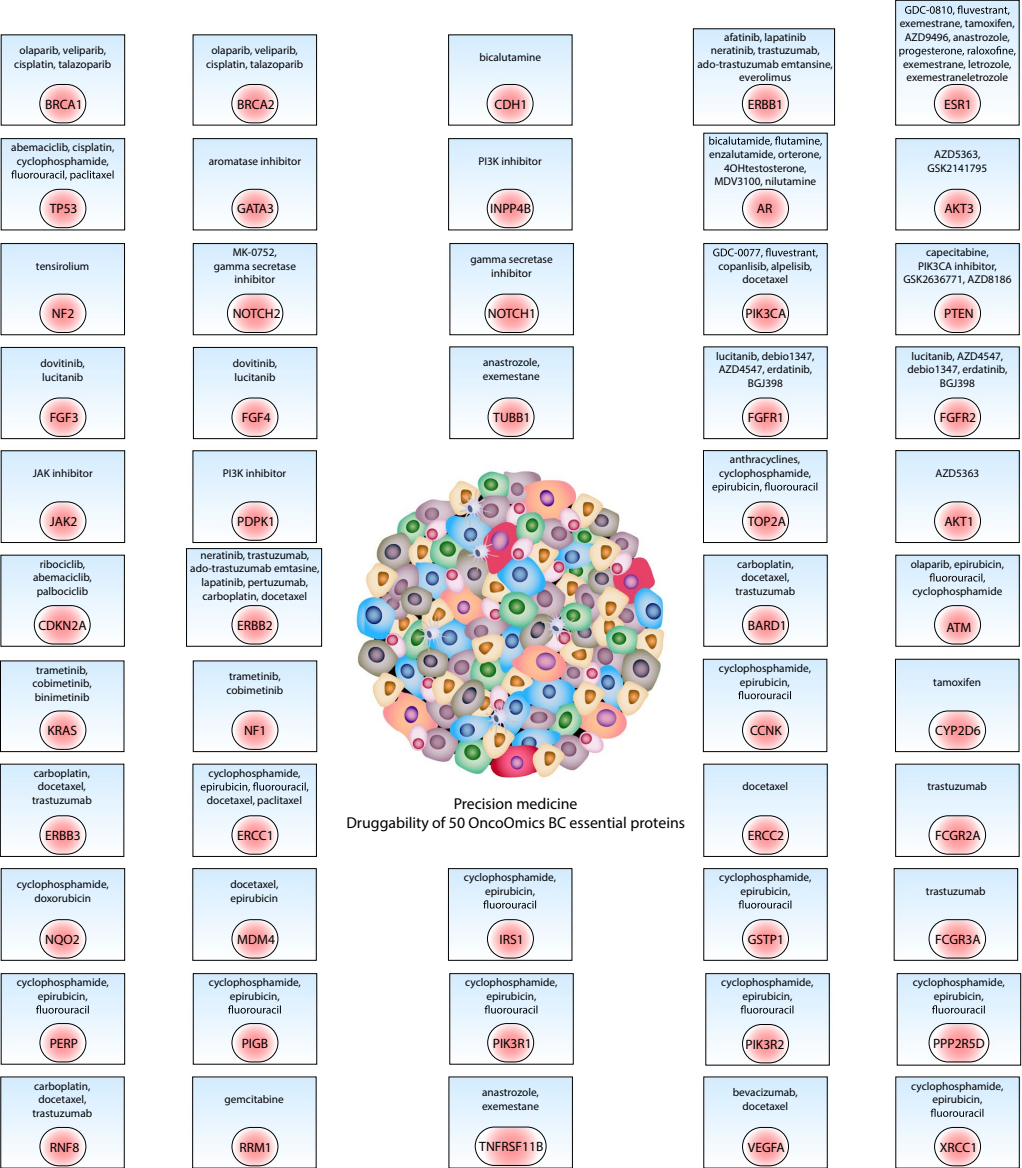
Precision medicine. Interaction between drugs and 50 OncoOmics BC essential proteins.

Consequently, based on the aforementioned somatic and germline oncogenic variants, the Cancer Genome Interpreter and PreMedKB platforms provided a comprehensive *in silico* list of biological therapy drugs aimed to improve precision medicine in breast cancer (Fig. 11, Tables S35 and S39).

## Discussion

In this study we reveal essential genes in breast cancer through an OncoOmics strategy that analyzes genomic alterations, PPi networking, protein expression, dependency maps and patient-derived xenografts in three gene sets. The first gene set was taken from our previous study where we developed a Consensus Strategy that was proved to be highly efficient in the recognition of BC pathogenic genes^29,41^. The second gene set was taken from several studies of PCA, which provides a panoramic view of the oncogenic processes that contributes to BC pathogenesis^3,13,31–37^. The third gene set was taken from the CGI and PharmGKB. On the one hand, the CGI flags genomic biomarkers of drug response with different levels of clinical relevance^38^. On the other hand, PharmGKB collects clinical annotations applied in BC patients and taken from the NCCN, ESMO, CPNDS, DPWG and CPIC guidelines^43–46^. Finally, the compendium of these 230 genes was analyzed through four different OncoOmics approaches.

The first OncoOmics approach consisted in the analysis of genomic alterations using the PCA data^47,48^. The frequency mean of genomic alterations in the CS (1.2) and PCA (1.3) gene sets were significantly higher than both the non-cancer genes (0.4) and the well-known BC driver genes (0.8), with a significant Bonferroni correction of P < 0.001. This means that the analyzed set of genes might be strongly associated with BC (Fig. 1a).

The most common genomic alterations in a cohort of 994 individuals were mRNA up-regulation, CNV amplification and missense mutations. Regarding molecular subtypes, basal-like showed the highest amount of genomic alterations. *PIK3CA* was the most altered gene in luminal A, *CCND1* in luminal B, *TP53* in basal-like and normal-like, and *ERBB2* in Her2-enriched (Fig. 2a). A multiple comparison through Bonferroni correction found significant differences (P < 0.05) of CNV amplifications, CNV deep deletions, mRNA up-regulations, and mRNA down-regulations among molecular subtypes (Figs. 2c–f). Regarding tumor stages, T2 showed the highest amount of genomic alterations. *PIK3CA* was the most altered gene in T1, *TP53* in T2 and T3, and *ERBB2* in T4 (Fig. 2g). Bonferroni correction found significant differences (P < 0.05) in punctual mutations, CNV amplifications, CNV deep deletions, mRNA up-regulations, and mRNA down-regulations among tumor stages (Fig. 2h–l). Lastly, the first OncoOmics approach revealed that 73 essential genes presented frequencies of alteration higher than the average (Fig. 3a)^3,13,31–37^.

Subsequently, the enrichment analysis of signaling pathways was carried on taking into account all genomic alterations in the 230 genes using David Bioinformatics Resource and KEGG^49,52^. Pathways with the highest amount of genomic alterations per molecular subtype were Jak-STAT in luminal A, Wnt in luminal B, p53 in basal-like, ERBB in Her2-enriched and Hippo in normal-like. Bonferroni correction showed significant differences (P < 0.05) among several subtypes as shown in Fig. 4b. On the other hand, pathways with the highest amount of genomic alterations per tumor stage were Wnt in T1, T2 and T3, and thyroid hormone in T4. Bonferroni correction showed significant differences (P < 0.05) comparing T1 with T2 and T4 as shown in Fig. 4d.

Regarding previously mentioned signaling pathways, Jak-STAT is involved in inflammatory response, stem cell maintenance, and hematopoiesis^81^. The Wnt signaling pathway actively functions in embryonic development and helps in homeostasis in mature tissues by regulating cell survival, migration, proliferation, and polarity^82^. The p53 signaling pathway plays an essential role into inhibition of growth, programmed cell death, cell migration and angiogenesis^83^. The ERBB pathway mediates signal transduction events that control cell survival, migration and proliferation in BC^84^. The Hippo pathway plays important roles in tumor suppression and immune response. However, alterations in this pathway are involved in the BC tumorigenesis and metastasis^85^. Lastly, the thyroid hormone pathway plays an important role as regulator of growth and metabolism. Nevertheless, dysfunction of the T3 hormone promotes cancer progression in mammary epithelial cells^86^.

The second OncoOmics approach was focused on proteins with the highest degree centrality and consensus score in the String PPi network. In accordance with Li *et al*. and Ivanov *et al*.^56,87^, PPi with therapeutic significance can be revealed by the integration of cancer proteins into networks. PPi regulate essential oncogenic signals to cell proliferation and survival, and thus, represents potential targets for drug development and drug discovery. Regarding our networking analysis, the final interaction network consisted in 258 nodes with a degree centrality average of 48.8 and a consensus score average of 0.803^29^; the sub-network integrated by 198 of 230 nodes had 52.7 of degree centrality and 0.812 of consensus scoring; finally, the sub-network integrated by 65 of 73 proteins with the highest amount of genomic alterations had 61.7 of degree centrality and 0.833 of consensus score. Hence, a sub-network of nodes with the highest amount of genomic alterations presented a highest degree centrality and consensus score, suggesting that there is strong correlation between these proteins and BC. Additionally, the oncogenomics validation showed a substantial correlation between our String PPi network (Fig. 5a) and the OncoPPi BC network (Fig. 5b), identifying 16 nodes strongly associated with BC^29^. The second OncoOmics approach revealed 40 essential proteins with the highest degree centrality and consensus scoring.

The third OncoOmics approach was focused on proteins with significant high and low expression in BC proteome. More than 500 proteins have been identified as strongly involved in oncogenesis. Loss of expression, overexpression or expression of dysfunctional proteins contribute to uncontrolled tumor growth, causing chromosomal rearrangements, gene amplification and ungoverned methylation^88^. Regarding our 230 proteins, 43 showed significant high (Z-scores ≥ 2) and low (Z-scores ≤ −2) expression according to TCGA^89^ (Fig. 6a); and 16 proteins showed opposite expression between healthy and affected tissues after microarray-based immunohistochemistry according to the Human Protein Altas (Fig. 6b)^57,58^. The compendium of 60 proteins with significant high and low expressions made up the third OncoOmics approach.

The fourth OncoOmics approach was related to the BC dependency map in cell lines and patient-derived xenografts. According to Tsherniak *et al*., mutations that trigger the growth of cancer cells also confer specific vulnerabilities that normal cells lack, and these dependencies are compelling therapeutic targets^19^. The cancer dependency map identifies essential genes in proliferation and survival of well-annotated cell lines through systematic loss-of-function screens^19–22^. On the one hand, DETEMER2 analyzed the genome-scale RNAi loss-of-function screens, and on the other hand, CERES analyzed the genome-scale CRISPR-Cas9 loss-of-function screens as shown in Fig. 7a. In addition to the loss-of-function screens in a large number of well-annotated BC cell lines, the patient-derived xenografts are *in vivo* models of human tumors engrafted in a mouse host and emerging as a powerful tool for understanding tumor hallmarks and predicting drug efficacy^90^. Consequently, we validated the genomic expression of the strongly selective and common essential genes (dependencies in BC cell lines) in breast tumors from PDXs provided by the Jackson Laboratory^59^. The fourth OncoOmics approach was made up of 38 essential proteins in BC (Fig. 7e).

Subsequently, the compendium of essential genes per approach reveals the 140 OncoOmics BC essential genes (Fig. 8a). *RAC1*, *AKT1*, *CCND1*, *PIK3CA* and *ERBB2* were essential genes in all the OncoOmics approaches. *CDH1*, *MAPK14*, *TP53*, *MAPK1*, *SRC* and *RAC3* showed genomic alterations, highest degree centrality and consensus scores in the String PPi network, and significant protein expression. *GRB2* showed genomic alterations, highest degree centrality and consensus scores in the String PPi network, and substantial relevance in BC cell lines and PDXs. *MED1* and *GATA3* showed genomic alterations, significant protein expression, and considerable relevance in BC cell lines and PDXs. Lastly, *BCL2*, *CTNNB1*, *EGFR* and *CDK2* showed significant protein expression, highest degree centrality and consensus scores in the String PPi network, and substantial relevance in BC cell lines and PDXs.

Relevant studies worldwide have identified OncoOmics BC essential genes. For instance, genome-wide association studies performed by the Breast Cancer Association Consortium showed that *BRCA2*, *CHEK2*, *ESR1*, *FGFR2*, *MDM4* and *PIK3R3* carry germline variants associated with BC development^74–77^. According to Bailey *et al*., identifying molecular cancer drivers is critical for precision oncology^32^. Their final consensus list was conformed by 29 BC driver genes, of them, 22 were OncoOmics BC essential genes (*AKT1*, *ARID1A*, *BRCA1*, *CASP8*, *CDH1*, *CDKN1B*, *CTCF*, *ERBB2*, *FOXA1*, *GATA3*, *KMT2C*, *KRAS*, *MAP2K4*, *MAP3K1*, *NCOR1*, *NF1*, *PIK3CA*, *PIK3R1*, *PTEN*, *RB1*, *SF3B1* and *TP53*). According to Gonzalez-Perez *et al*., the IntOGen-mutation platform summarizes somatic mutations involved in tumorigenesis^91^. Their final consensus list was conformed by 99 mutational BC driver genes, of them, 34 were identified by the OncoOmics strategy (*TP53*, *PIK3CA*, *KMT2C*, *GATA3*, *CDH1*, *MAP3K1*, *ESR1*, *PTEN*, *AKT1*, *NCOR1*, *ARID1A*, *MAP2K4*, *FOXA1*, *NF1*, *ERBB2*, *RB1*, *SF3B1*, *ERBB3*, *CTCF*, *PIK3R1*, *ATM*, *FGFR2*, *BRCA1*, *CASP8*, *CREBBP*, *BRCA2*, *CDKN2A*, *KRAS*, *CDKN1B*, *NOTCH2*, *MAX*, *MDM4*, *EGFR* and *JAK2*). Finally, the PCAWG Consortium of the International Cancer Genome Consortium (ICGC) and The Cancer Genome Atlas reported an integrative analysis of 2,658 whole-cancer genomes across 38 tumor types^92^. Regarding breast cancer, PCAWG identified 27 mutational BC driver genes, of them, 15 were OncoOmics BC essential genes (*TP53*, *PIK3CA*, *MAP3K1*, *KMT2C*, *NOTCH2*, *SF3B1*, *PTEN*, *ARID1A*, *MAP2K4*, *AKT1*, *CTCF*, *FOXA1*, *RB1*, *CDKN2A* and *ATM*).

According to Reimand *et al*., g:Profiler lets us know the enrichment map of the 140 OncoOmics BC essential genes^66^. The most significant GO: biological process was the positive regulation of macromolecule metabolic process, the GO: molecular function was phosphatidylinositol 3-kinase activity, the Reactome pathway was generic transcriptor pathway, and the most significant Human Phenotype Ontology term was breast carcinoma^68^. Subsequently, the most relevant network interactions of the GO: biological process and the Reactome pathways were related to immune system, tyrosine kinase, cell cycle and DNA repair terms (Figs. 9 and S2)^54,66^.

There is currently great enthusiasm about immunotherapeutic strategies to treat BC^93^. The first approval of an immune checkpoint blockade agent for treatment of BC came in March 2019 when the anti-PD-L1 antibody atezolizumab was approved to be used with nab-paclitaxel in triple-negative BC patients^94,95^. 16 OncoOmics BC essential genes were associated with immunotherapy^61,96^ as shown in Fig. 8C. Kinases have been recognized as therapeutic targets due to their druggability and play a critical role in cell migration, differentiation, growth and survival^97^. 15 OncoOmics BC essential genes were kinomes^62^. Cell cycle comprises a series of events that drive cell division and DNA replication^98^. 12 OncoOmics BC essential genes were involved in cell cycle^63^. DNA repair signaling pathways work in concert to correct DNA lesions and maintain genome stability. Nevertheless, a defective DNA repair machinery causes BC development and progression^99^. 17 OncoOmics BC essential genes were involved in DNA repair^64^. RBPs are key players in post-transcriptional events and are emerging as critical modulators in BC^100–102^. Bioinformatics profiling of tumors have revealed the landscape of alterations in RBPs across cancer types^103–106^. Lastly, 10 OncoOmics BC essential genes were RBPs^65^.

Regarding clinical trials reported on the OncoOmics BC essential proteins, the Open Targets Platform is an available resource for the integration of genomics and chemical data to aid systematic drug target identification and prioritization^69^. There are 98 drugs that are being analyzed in 2,904 clinical trials in 28 of 140 OncoOmics BC essential proteins. Additionally, there are 30 drugs involved in 736 clinical trials in phases 3 and 4. The top five drugs with the highest number of clinical trials in process or completed are paclitaxel (111), docetaxel (105), trastuzumab (80), tamoxifen (69), and doxorubicin (60)^69^ (Fig. 10e).

Tumor-related genomic alterations predict tumor prognosis, drug response, and toxicity^107^. Precision medicine provides patients with the most appropriate diagnostics and targeted therapies based on the ‘omics’ profile and other predictive and prognostic tests^108^. Therefore, precision medicine aims to deliver the right medicine to the right patient at the right dose at the right time, minimizing adverse effects and maximizing drug efficacy^109,110^. Figure 11 shows comprehensive interactions between directed biological drugs and 50 OncoOmics BC essential proteins aimed to improve precision medicine in breast cancer.

In conclusion, since BC is a complex and heterogeneous disease, the study of different OncoOmics approaches is an effective way to reveal essential genes to better understand the molecular landscape of processes behind oncogenesis, and to develop better therapeutic treatments focused on pharmacogenomics and precision medicine.

## Methods

### OncoPrint of genomic alterations according to the Pan-Cancer Atlas

PCA has reported the clinical data of 1084 individuals with BC and it can be visualized in the Genomic Data Commons of the National Cancer Institute (https://gdc.cancer.gov/) and in the cBioPortal (http://www.cbioportal.org/)^47,48^. The clinical annotations were age, pTNM classification, tumor type, tumor stage and race/ethnicity.

Additionally, PCA has reported genomic alterations (mRNA up-regulation, mRNA down-regulation, CNV amplification, CVN deep deletion, putative driver mutations and fusion gene) of 994 individuals. Putative mutations were analyzed through exome sequencing, CNVs through the Genomic Identification of Significant Targets in Cancer (GISTIC 2.0)^111,112^, and mRNA expression through RNA Seq V2. We analyzed five gene sets in order to compare the frequency mean of genomic alterations among them. The first gene set (n = 177) was integrated by the non-cancer genes^113^. We calculated the OncoScore of non-cancer genes, taking out all genes from our study. The second gene set (n = 119) was the BC driver genes, according to The Network of Cancer Genes^60^. The third gene set (n = 84) was taken from our previous study where we developed a Consensus Strategy of prioritized genes related to BC pathogenesis^29^. The fourth gene set (n = 85) was made up of genes associated with BC development, according to several PCA studies^31,32,114^. Finally, the fifth gene set (n = 91) consisted of BC biomarkers and druggable enzymes taken from PharmGKB and the CGI (Supplementary Table S2)^38,39,42^.

The OncoOmics approaches were performed in 230 genes conformed by the CS, PCA and PharmGKB/CGI gene sets. We calculated the percentage and ratio of genomic alterations per intrinsic molecular subtype and tumor stage, and then we established a ranking of genes with the highest amount of genomic alterations (OncoPrint). The OncoPrint conformed the first OncoOmics approach.

### Pathway enrichment analysis

The enrichment analysis of signaling pathways was performed using David Bioinformatics Resource to obtain integrated information from KEGG^49–52^. It was carried on in the 230 genes, taking into account terms with a significant FDR < 0.01. After that, genomic alterations that comprise each signaling pathway were analyzed, taking into account the molecular subtype and tumor stage of individuals from PCA. Circos plots and violin plots were designed to visualize all data. Lastly, in order to compare the ratio of genomic alterations among subtypes and tumor stages, normalization was carried out dividing the number of genomic alterations by the number of individuals per subtype and tumor stage. Regarding molecular subtypes, 499 individuals were luminal A, 197 were luminal B, 171 were basal-like, 78 were Her2-enriched and 36 were normal-like, and regarding tumor stage, 255 were T1, 586 were T2, 113 were T3, and 103 were T4.

### Protein-protein interactome network

The PPi network with a highest confidence cutoff of 0.9 and zero node addition was created using the String Database, which takes into account predicted and known interactions^53^. The confidence scoring is the approximate probability that a predicted link exists between two enzymes in the same metabolic map, whereas the degree centrality of a node means the number of edges the node has to other nodes in a network. The centrality indexes calculation and network visualization were analyzed through the Cytoscape software^54^. Proteins with the highest degree centrality, consensus score and sub-networks were differentiated by colors in the PPi network. On the other hand, OncoPPi (http://oncoppi.emory.edu/) reports the development of a cancer-focused PPi network, identifying more than 260 high-confidence cancer-associated PPi^55,56^. In addition, the OncoPPi BC network consisted of 16 proteins and 18 PPi experimentally analyzed in BC cell lines^55,56^. The correlation of the degree centrality by means of Spearman P-value test between our String PPi network and the OncoPPi BC network allowed for the validation of all the high-confidence BC-focused PPi analyzed in cell lines^29^. Lastly, proteins with the highest degree centrality and consensus scoring made up the second OncoOmics approach.

### Protein expression analysis

TCGA has reported the protein expression data of 994 individuals with BC through RPPA and mass spectrometry by the Clinical Proteomic Tumor Analysis Consortium (CPTAC), and it can be visualized in the cBioPortal^47,48^. We analyzed the protein expression of 230 protein where Z-scores ≥ 2 mean a significant high protein expression and Z-scores ≤ −2 mean a significant low protein expression.

On the other hand, the Human Protein Atlas (https://www.proteinatlas.org/) explains the diverse molecular signatures of proteomes in human tissues based on an integrated ‘omics’ approach that involves quantitative transcriptomics and tissue microarray-based immunohistochemistry^58,88,115^. We compared the protein expression levels (high, medium, low and non-detected) of our 230 proteins between normal and BC tissues. Finally, all genes with the altered protein expression made up the third OncoOmics approach.

### Breast cancer dependency map

The DepMap project (https://depmap.org/portal/) is collaboration between the Broad Institute and the Welcome Sanger Institute. Multiple genetic or epigenetic changes provide cancer cells with specific vulnerabilities that normal cells lack. Even though the landscape of genomic alterations has been extensively studied to date, we have limited understanding of the biological impact of these alterations in the development of specific tumor vulnerabilities, which triggers a limited use of precision medicine in the clinical practice worldwide. Therefore, the main goal of DepMap is to create a comprehensive preclinical reference map connecting tumor features with tumor dependencies to accelerate the development of precision treatments^19–22^.

In order to identify essential genes for BC cell proliferation and survival, DepMap performed systematic loss-of-function screens in a large number of well-annotated BC cell lines representing the tumor heterogeneity and their molecular subtypes. The DEMETER2 algorithm was applied to analyze genome-scale RNAi loss-of-function screens in 73 BC cell lines and 711 cancer cell lines, whereas the CERES algorithm was applied to analyze genome-scale CRISPR-Cas9 loss-of-function screens in 28 BC cell lines and 558 cancer cell lines^20,22^. In addition to existing cell lines, the Cancer Cell Line Encyclopedia (CCLE) project will greatly expand the collection of characterized cell lines to improve precision treatments^116^.

Regarding dependency scores, a lower score means that a gene is more likely to be dependent in a specific cancer cell line. A score of 0 means that a gene is not essential, whereas a score of −1 corresponds to the median of all common essential genes. A strongly selective gene means that its dependency is at least 100 times more likely to have been sampled from a skewed distribution than a normal distribution. A common essential gene is when in a pan-cancer screen its gene ranks in the top most depleting genes in at least 90% of cell lines^19^. All genes or proteins with a dependency score ≤ −1 were subsequently analyzed with patient-derived xenografts.

### Patient-derived xenografts

The Jackson Laboratory PDX resource (http://tumor.informatics.jax.org/mtbwi/pdxSearch.do) comprises 455 PDX models originating from 34 different primary sites^59^. Even though, we analyzed expression levels of strongly selective and common essential proteins in breast cancer obtained from the analysis of BC dependency map in cell lines. Significant high protein expression has a Z-score ≥ 2 and significant low protein expression has a Z-scores ≤ −2.

### Enrichment map of the OncoOmics BC essential genes

The pathway enrichment analysis gives scientists curated interpretation of gene lists generated from genome-scale experiments^66^. The OncoOmics essential genes in BC were analyzed by using g:Profiler (https://biit.cs.ut.ee/gprofiler/) in order to obtain significant annotations (FDR < 0.001) related to GO terms, pathways, networks and disease phenotypes. Subsequently, g:Profiler annotations were analyzed with the EnrichmentMap software in order to generate network interactions of the most relevant GO: biological processes and Reactome pathways, and these networks were visualized using Cytoscape^54,66^.

### Clinical trials

The Open Targets Platform (https://www.targetvalidation.org) is comprehensive and robust data integration for access to and visualization of drugs involved in clinical trials associated with BC proteins, detailing its phase, status, type and target class^69^. In addition, we created a Sankey plot to better understand which drugs are involved in the most advanced phases (3 and 4) of clinical trials.

### Precision medicine

Precision oncology focuses on matching the most effective treatment based on the ‘omics’ profile of each individual or population^70,71^. The CGI (https://www.cancergenomeinterpreter.org/home) flags genomic biomarkers of drug response with different levels of clinical relevance^38^. Huang *et al*. and the Pan-Cancer Atlas project conducted the largest investigation of pathogenic germline variants in cancer^73^. Long *et al*.^74,75^, Cai *et al*.^76^, and Michailidou *et al*.^77^, performed genome-wide association studies identifying germline variations related to BC development. PreMedKB (http://www.fudan-pgx.org/premedkb/index.html#/home) is a bioinformatics tool that facilitates the interpretation of the clinical meaning of a patient's genetic variants^71^. PharmGKB (https://www.pharmgkb.org/) collected complete guidelines for application of pharmacogenomics in clinical practice, according to several consortiums worldwide^43–46^. Finally, PCAWG Consortium (https://dcc.icgc.org/) revealed an integrative analysis of genomic alterations in coding and non-coding regions^6,92^.

Based on the aforementioned somatic and germline oncogenic variants we performed two analyses. On the one hand, we analyzed the consequence type of variants with the Ensembl Variant Effector Predictor (https://www.ensembl.org/Multi/Tools/VEP?db=core), which is a powerful toolset for the annotation of genomic variants in coding and non-coding regions^80^. On the other hand, we analyzed oncogenic variants through the Cancer Genome Interpreter and PreMedKB platforms to provide a comprehensive *in silico* list of biological therapy drugs^38,71^.

### Statistical analyses

We performed a multiple comparison using the Bonferroni correction test (significant level of P < 0.05 and a 95% confidence interval) to analyze: 1) significant differences of genomic alteration frequencies among non-cancer genes, BC driver genes, Consensus Strategy, Pan-Cancer Atlas and PharmGKB/CGI genes; 2) significant differences of genomic alteration frequencies among intrinsic molecular subtypes and tumor stages; 3) significant differences of genomic alteration frequencies of signaling pathways among molecular subtypes and tumor stages. A significant correlation of the degree centrality between the String PPi network and the OncoPPi BC network was performed using the Spearman p-value test with a P < 0.05. The significant high and low protein expression in humn tissues and patient-derived xenografts was considered using the Z-score. Z-score **≥** 2 means significant high protein expression and Z-scores ≤ −2 means significant low protein expression. Lastly, the enrichment map of OncoOmics BC essential genes was performed using g:Profiler that determines the most significant GO: biological processes, GO: molecular functions, Reactome pathways, WikiPathways, KEGG pathways and human phenotype ontology with a false discovery rate <0.001.

## Supplementary information

Supplementary Information.

Supplementary Dataset.

## Data Availability

All data generated or analyzed during this study are included in this published article (and its Supplementary Information files).
