# Supplementary material for: OncoOmics approaches to reveal essential genes in breast cancer: a panoramic view from pathogenesis to precision medicine
Source: Sci Rep. 2020 Mar 24;10:5285. doi: 10.1038/s41598-020-62279-2 (PMC7093549; doi:10.1038/s41598-020-62279-2)
Supplement: Supplementary file 1 — Supplementary Information. [file 41598_2020_62279_MOESM1_ESM.pdf]

OncoOmics approaches to reveal essential genes in breast cancer: a panoramic view from pathogenesis to precision medicine

Andrés López-Cortés,[1,2,3*] César Paz-y-Miño,[1] Santiago Guerrero,[1] Alejandro Cabrera-Andrade,[2,4,5] Stephen J. Barigye,[6] Cristian R. Munteanu,[2,7,8] Humberto González-Díaz,[9,10] Alejandro Pazos,[2,7,8] Yunierkis Pérez-Castillo,[5,11] and Eduardo Tejera[5,12,*]

1 Centro de Investigación Genética y Genómica, Facultad de Ciencias de la Salud Eugenio Espejo, Universidad UTE, Mariscal Sucre Avenue, Quito 170129, Ecuador

2 RNASA-IMEDIR, Computer Science Faculty, University of A Coruna, A Coruna 15071, Spain

3 Red Latinoamericana de Implementación y Validación de Guías Clínicas Farmacogenómicas (RELIVAF-CYTED)

4 Carrera de Enfermería, Facultad de Ciencias de la Salud, Universidad de Las Américas, Avenue de los Granados, Quito 170125, Ecuador

5 Grupo de Bio-Quimioinformática, Universidad de Las Américas, Avenue de los Granados, Quito 170125, Ecuador

6 Department of Chemistry, McGill University, 801 Sherbrooke Street West, Montreal, QC H3A 0B8, Canada

7 Biomedical Research Institute of A Coruña (INIBIC), University Hospital Complex of A Coruna (CHUAC), A Coruna 15006, Spain

8 Centro de Investigación en Tecnologías de la Información y las Comunicaciones (CITIC), Campus de Elviña s/n, A Coruna 15071, Spain

9 Department of Organic Chemistry II, University of the Basque Country UPV/EHU, Leioa 48940, Biscay, Spain

10 IKERBASQUE, Basque Foundation for Science, Bilbao 48011, Biscay, Spain

11 Escuela de Ciencias Físicas y Matemáticas, Universidad de Las Américas, Avenue de los Granados, Quito 170125, Ecuador

[12] Facultad de Ingeniería y Ciencias Agropecuarias, Universidad de Las Américas, Avenue de los Granados, Quito 170125, Ecuador

*** Authors to whom correspondence should be addressed**

Andrés López-Cortés, MSc.

Centro de Investigación Genética y Genómica, Facultad de Ciencias de la Salud Eugenio Espejo, Universidad UTE, Mariscal Sucre Avenue, Quito 170129, Ecuador. E-mail: aalc84@gmail.com

Eduardo Tejera, PhD.

Facultad de Ciencias de la Salud, Universidad de Las Américas, Avenue de los Granados, Quito 170125, Ecuador. E-mail: eduardo.tejera@udla.edu.ec

Supplementary Figure S1

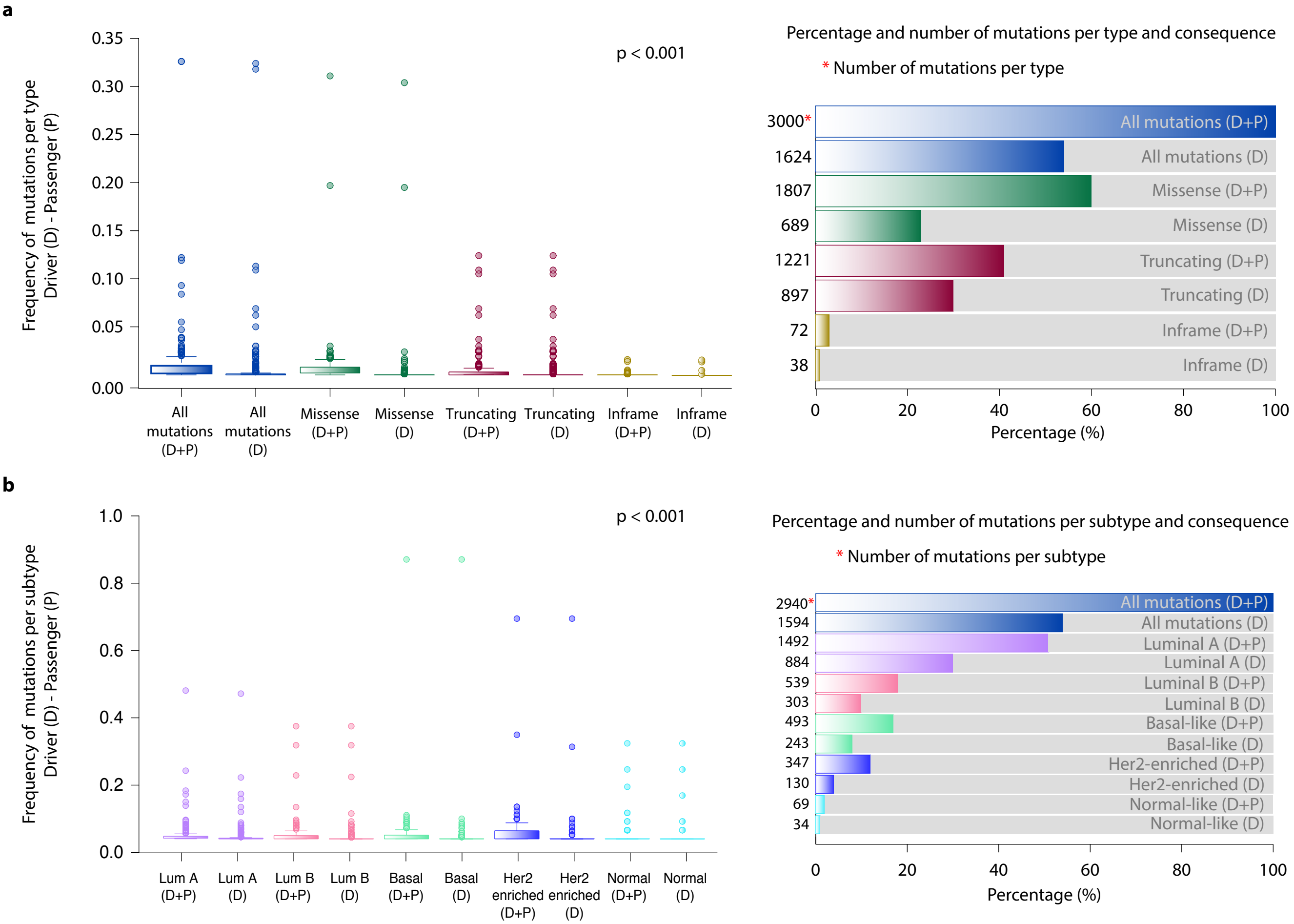

Supplementary Figure S1 | a) Frequency of mutations per type. b) Frequency of mutations per molecular subtype.

Supplementary Figure S2

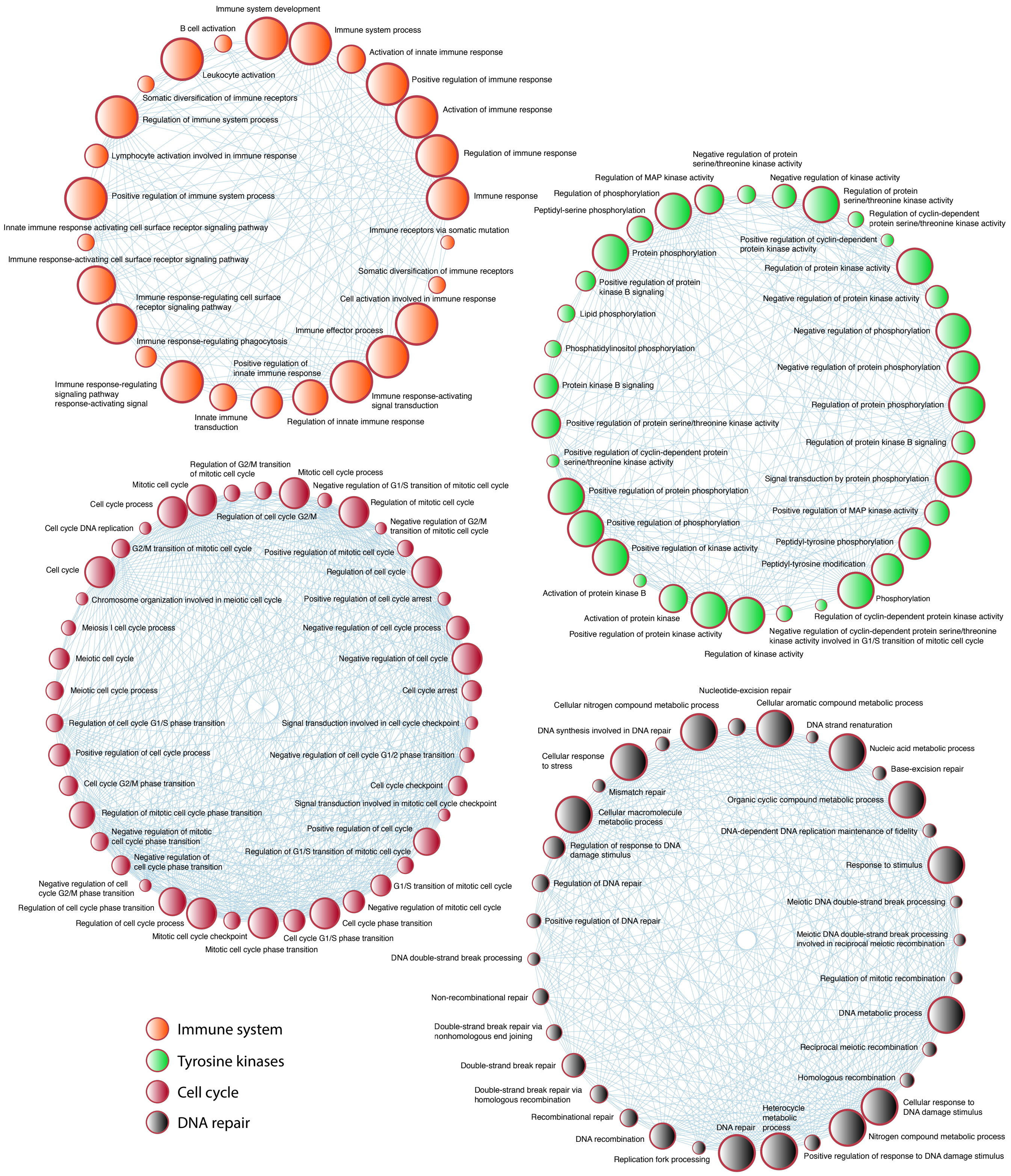

Supplementary Figure S2 | Network interactions of the most significant GO: biological processes.
